# Worldwide transmission and infection risk of mosquito vectors of West Nile, St. Louis encephalitis, Usutu and Japanese encephalitis viruses: a systematic review

**DOI:** 10.1038/s41598-022-27236-1

**Published:** 2023-01-06

**Authors:** María José Tolsá-García, Magdalena Laura Wehmeyer, Renke Lühken, David Roiz

**Affiliations:** 1 MIVEGEC, IRD, CNRS, Université Montpellier, Montpellier, France; 2 International Joint Laboratory ELDORADO, IRD/UNAM, Mexico City, Mexico; 3 Fauna Silvestre y Animales de Laboratorio, Departamento de Etología, Facultad de Medicina Veterinaria y Zootecnia, UNAM, Mexico city, Mexico; 4 Bernhard Nocht Institute for Tropical Medicine, Hamburg, Germany

**Keywords:** Ecological epidemiology, Macroecology, Entomology, Viral vectors, West nile virus

## Abstract

The increasing trend of mosquito-borne pathogens demands more accurate global estimations of infection and transmission risks between mosquitoes. Here, we systematically review field and laboratory studies to assess the natural field infection and experimental laboratory transmission risk in *Culex* mosquitoes. We studied four worldwide flaviviruses: West Nile, Usutu, Japanese encephalitis, and St. Louis encephalitis, belonging to the Japanese encephalitis Serocomplex (JES). The PRISMA statement was carried out for both approaches. The Transmission-Infection Risk of the diverse mosquito species for the different viruses was estimated through seven variables. We considered 130 and 95 articles for field and experimental approach, respectively. We identified 30 species naturally infected, and 23 species capable to transmit some of the four flaviviruses. For the JES, the highest Transmission-Infection Risk estimate was recorded in *Culex quinquefasciatus* (North America). The maximum Infection-Transmission Risk values for West Nile was *Culex restuans*, for Usutu it was *Culex pipiens* (Europe), for St. Louis encephalitis *Culex quinquefasciatus* (North America), and for Japanese encephalitis *Culex gelidus* (Oceania). We conclude that on a worldwide scale, a combination of field and experimental data offers a better way of understanding natural infection and transmission risks between mosquito populations.

## Introduction

During recent decades the world has seen an increase in the emergence, incidence, and distribution of mosquito-borne viruses such as dengue, chikungunya, Zika, yellow fever, Usutu, West Nile, Japanese encephalitis, and Mayaro. These viruses have significant negative impacts on public health, economies, and wildlife conservation^1,2^. They can also spread into new geographic areas. Their major drivers of emergence include land use changes, international travel and commerce, human demographics, urbanisation, and climate change^3^.

Several of the mosquito-borne viruses found throughout the world are flaviviruses belonging to the Japanese encephalitis serocomplex (JES). Typical members of this group in terms of their consequences for public health and the conservation of wildlife are the Japanese encephalitis, Usutu, St. Louis encephalitis and West Nile viruses^4–6^. However, understanding their various transmission cycles is a challenge because of their enormous complexity. These viruses are mainly transmitted by numerous *Culex (Cx)* mosquito species and may be amplified by a wide range of vertebrates in enzootic, epizootic or epidemic cycles^7^.

Understanding the risk of transmission by the different *Cx.* mosquito vectors is crucial if efficient strategies to prevent and control the spread of mosquito-borne viruses are to be developed^8^. The overall aim was to estimate the risk of a particular *Cx.* spp. becoming naturally infected and transmitting a particular virus belonging to the JES. The first method involves measuring the minimum infection rate (MIR), which is calculated as the number of detected positive catches in a given trap-day, divided by the number of analyzed mosquitoes in the same trap-day, multiplied by 1000^9^. MIR values and their environmental contexts are important sources of information on the potential interactions between mosquito species and mosquito-borne viruses^9,10^.

The second method involves measuring vectorial capacity, which is calculated from entomological parameters, such as vector competence, vector density, survival rate, feeding preferences, microbiota and the extrinsic incubation period^11^. Vector competence is an empirically obtained laboratory measure that assesses the susceptibility of an arthropod vector to become infected and subsequent ability to transmit the pathogen. It is highly influenced by temperature simulated under laboratory conditions, and varies among species and populations under optimal environmental conditions^11–14^. Vector competence is calculated from transmission rates and transmission efficiency (TE), both of which are estimated through a process of infection, dissemination and transmission (saliva infection)^11,13,15^.

The viral infection in the body parts of the mosquito indicates their transmission potential. The virus transmission cycle is complete when the infective virions present in the mosquitos saliva are released into a vertebrate host as the mosquito takes blood meals^11^. In field studies, it is important to analyze the body parts of mosquito species independently, so that if the viral genome is detected in the body and legs only, it can consider them to unable to continue the virus transmission. There are currently few field studies on the dissemination in the different parts of the body of mosquitoes. Usually mosquitoes are tested complete in groups of 1–50 with whole bodies^14^.

However, because laboratory studies do not reflect environmental factors, such as fluctuations in local temperature, age of the mosquito, viral dose, virus strains and midgut microbiota^16^. It has recently been suggested that macroecological methods could help identify large-scale spatial and/or temporal patterns between hosts and parasites^7,17^. This would allow comparisons of the natural infection and transmission rates of flaviviruses among mosquito species^18^.

Macroecological approaches can be used to identify critical areas with the highest potential for virus introduction and circulation^19^. Furthermore, determining risk areas based on the distribution of key mosquito species and viruses provides a basis for targeted surveillance and vector control programmes^20^. To understand the global situation, more accurate estimates of the virus-vector interface, the distribution and ecological niche of mosquitoes are urgently needed^2^.

We therefore systematically reviewed a range of field and experimental studies in order to analyse the transmission potential of *Cx.* spp. mosquitoes for four flaviviruses belonging to the JES: Japanese encephalitis virus (JEV), St. Louis encephalitis virus (SLEV), Usutu virus (USUV) and West Nile virus (WNV). The overall aim was to estimate the risk of a particular *Cx.* spp. becoming naturally infected and transmitting a particular virus belonging to the JES under experimental conditions. The specific aims were: (i) to estimate natural infection rates based on field data; (ii) to estimate the different transmission rates of *Cx. mosquito* populations based on vector competence studies; and (iii) to determine the infection-transmission risk (ITR) based on the association between field infection rates and experimental transmission rates.

## Results

### Field approach

**Figure 1:**
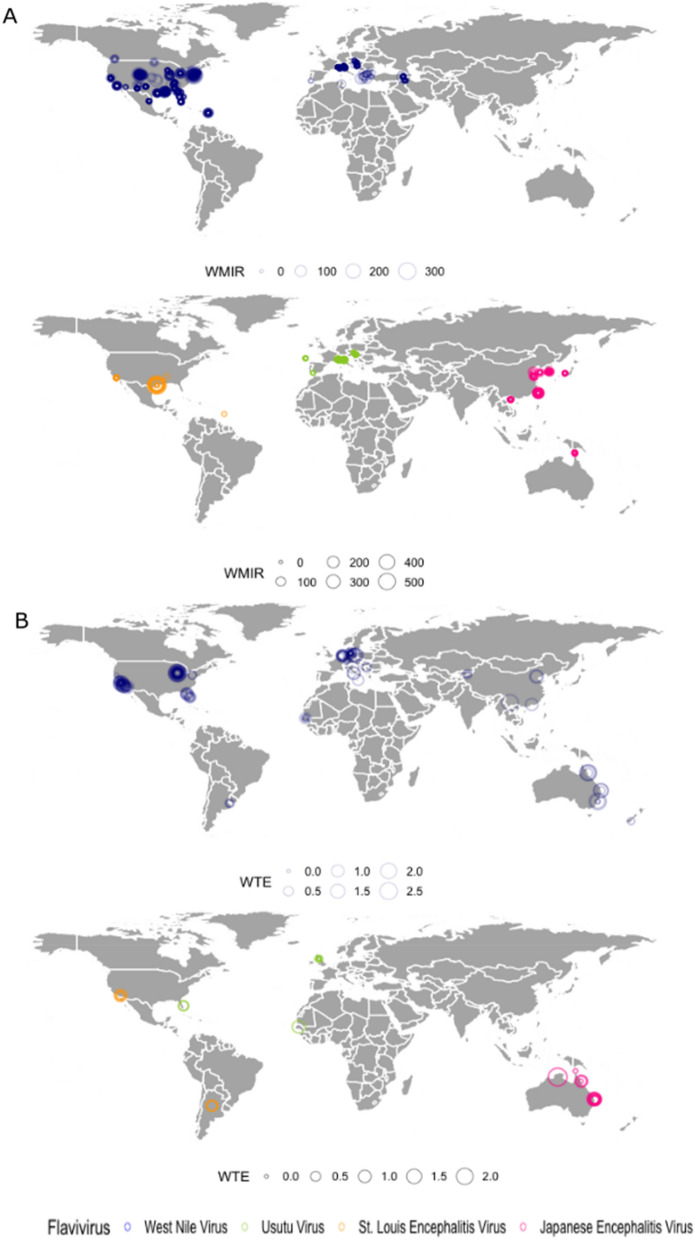
(**A**) Weighted Minimum Infection Rates and (**B**) Weighted Transmission Efficiency of mosquito populations for JES. High-quality data. The size of circles represents the magnitude of the estimates. Map was generated using R software version 4.1.2 with the packages mapdata, maps and tydiverse (https://www.r-project.org) and edited with Inkscape (https://inkscape.org/es/).

Our searches uncovered 301 papers reporting field studies. After screening the titles abstracts, and full texts, we kept 130 articles for the analysis (Supplementary Fig. 1), from which we obtained 1342 observations regarding 57 *Cx*. mosquito species from 28 countries and 135 localities (Fig. 1A). Of these 1342 observations, 733 (54.61%) were classified as high quality, (i.e., the number of individuals tested was specified) (Supplementary Tables 1 and 2). The best represented countries were the USA (64.7%, number of observations = 869), Italy (9.3%, n = 125), and Iran (2.9%, n = 39). Based on mosquito field surveillance and individuals testing positive, we concluded that JES is distributed mainly in the Nearctic, Palearctic and Oriental regions (Fig. 1A).

#### West Nile virus

WNV was detected mainly in the USA (76.5%, number of observations = 826), Italy (4.9%, n = 53) and Iran (3.6%, n = 39) (Fig. 1A). We also recorded 23 species (57%, 41 species) interacting with this virus (Supplementary Tables 1 and 3). *Cx. quinquefasciatus* became naturally infected in North America [Infection Frequency (IF) = 2.33] (Table 1). We recorded WNV interacting with *Cx. tritaeniorhynchus* in Asia (IF = 1.02), with *Cx. pipiens* in Europe (IF = 1.74) and with *Cx. antennatus, Cx. neavei, Cx. perexiguus, Cx. perfuscus, Cx. poicilipes, Cx. quinquefasciatus* and *Cx. tritaeniorhynchus* in Africa (IF = 1) (Supplementary Table 3).

**Table 1:** Description of the variables.

Variable	Description	Data type	Importance
**Field approach**			
Infection frequency (IF)	Represents the natural infection of mosquitos in natural conditions $IF = \frac{times\,\,positive}{{times \,\,tested\,\, by \,\,species}} \times \left( {1 + log10} \right)$	Discrete	This variable indicates the receptivity of mosquitos to natural infection
Standardized minimum infection rate (SMIR)	The MIR is the ratio of the number of positive pools to the total number of mosquitoes in the sample. It is the minimum infection proportion, and it assumes that only one infected individual is present in a positive pool^38^	Continuous	Represents the magnitude of the natural infection. If the virus is detected in dissected mosquitoes. However, it does not necessary represents an infection in the salivary glands
Infection risk (IR)	Represents the infection potential based on IF and SMIR $IR = IF \times SMIR$	Continuous	It combines the frequency and magnitude of a mosquito species to become naturally infected with the virus in the field
**Experimental approach**			
Transmission Frequency (TF)	Represents the presence of the virus in the mosquito saliva after an experimental infection $TF = \frac{times \,\,positive}{{times\,\, tested\,\, by\,\, species}} \times \left( {1 + log10} \right)$	Discrete	It shows the ability of each mosquito species to excrete the viruses every time infected. But it does not represent a magnitude of potential
Standardized transmission efficiency (STE)	TE, showing the proportion of individuals with infectious saliva among all individuals infected^15^	Continuous	The variable indicates the capacity of a mosquito to transmit the virus. But does not reflect the viral titer and the infectivity of viral particles presents in the saliva
Transmission risk (TR)	Describes the transmission potential based on the product of TF and the STE $TR = TF \times STE$	Continuous	It combines de frequency and magnitude of a mosquito species to transmit the virus in experimental conditions
Infection-transmission risk (ITR)	Represent the total risk based on the field and laboratory data	Continuous	It considers the transmission risk in experimental conditions and the natural infection risk

**Figure 2:**
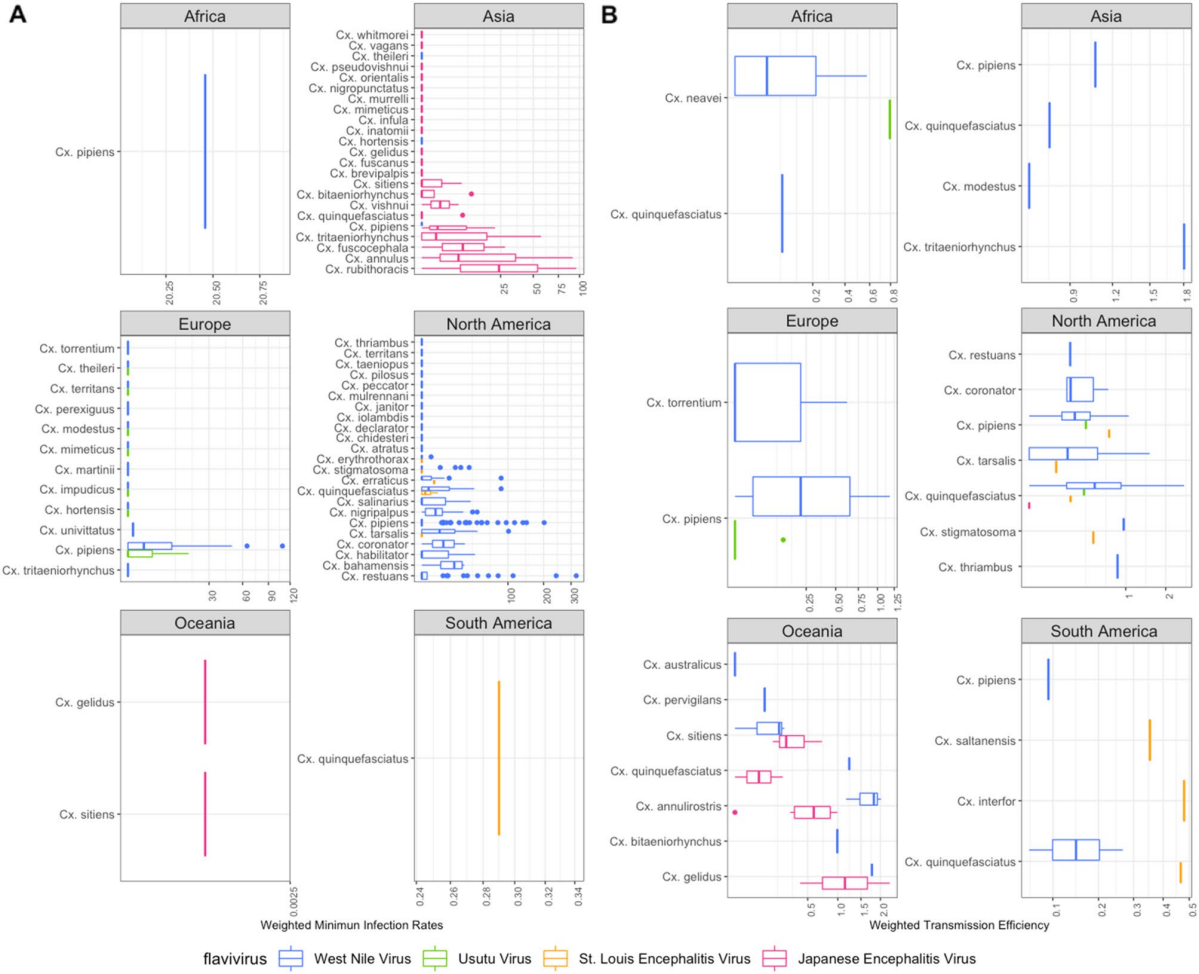
(**A**) Box plots for the Weighted Minimum Infection Rates and (**B**) Weighted Transmission Efficiency Rates for JES. Boxes indicate 2nd and 3rd quartiles, vertical lines upper and lower quartiles, and horizontal lines the median. Points indicate outliers. The Y axis was transformed to Sqrt (Square root).

**Figure 3:**
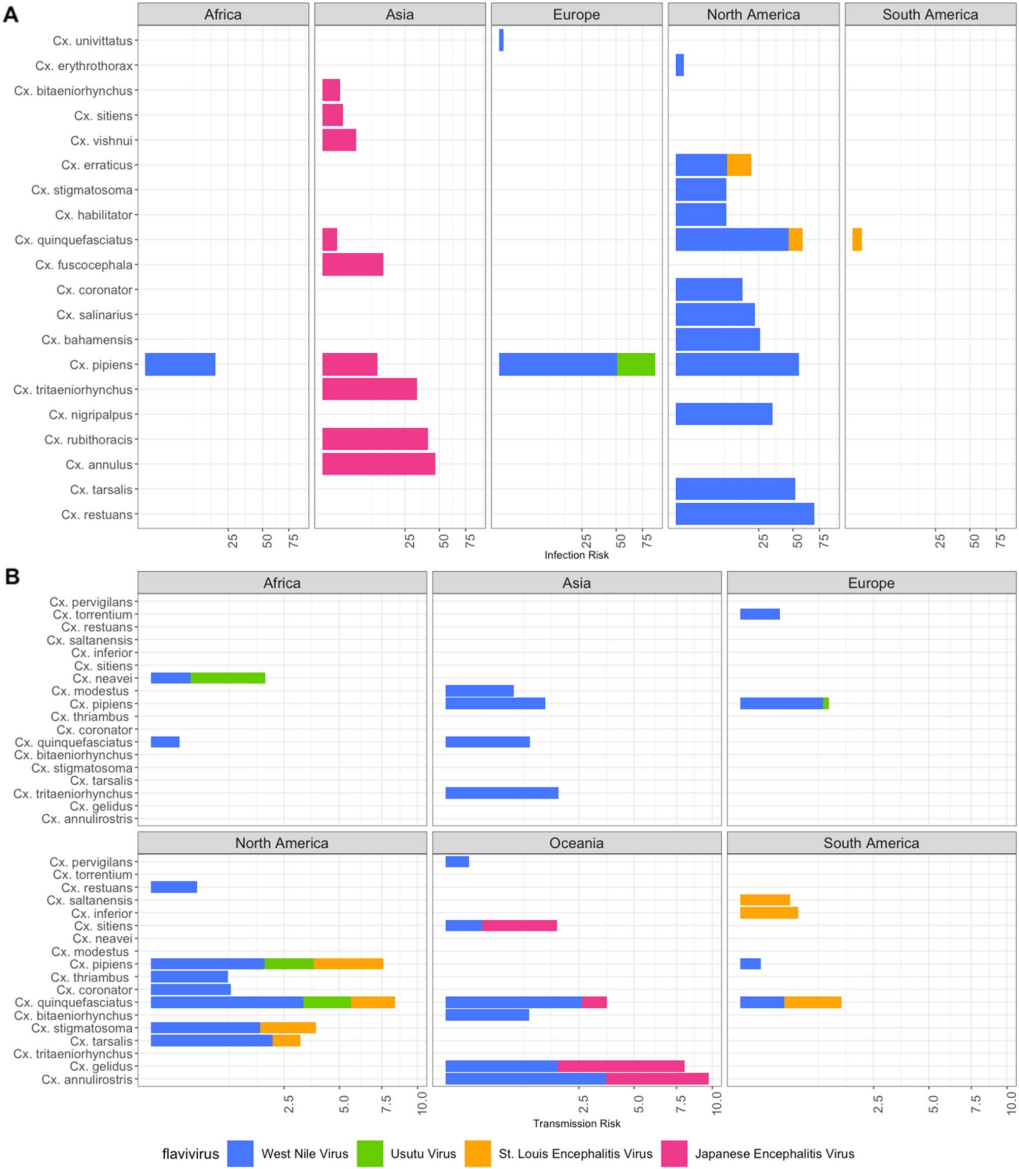
JES (**A**) Infection Risk and (**B**) Transmission Risk by mosquito species.

The highest infection rates were found in North America in *Cx. restuans* [Standardized minimum infection rate (SMIR) = 56.01], and in Africa and Europe in *Cx. pipiens* (SMIR = 20.45 and 29.25, respectively). No positive SMIR values were reported in Asian mosquitoes, and Oceanic mosquitoes were not sampled for this virus (Fig. 2A and Supplementary Table 3). The highest infection risk or potential was recorded in species from the USA, such as *Cx. restuans* (Infection Risk (IR) = 69.50), *Cx. pipiens* (IR = 55) and *Cx. tarsalis* (IR = 52.16) (Fig. 3A and Supplementary Table 3). Finally, WNV lineage 1 was detected in Algeria, Turkey, Portugal, Mexico, Tunisia, Iran, Spain and Italy, lineage 2 in Italy, Bulgaria, Greece and the Czech Republic, and lineage 5 in India (Supplementary Table 2).

#### Japanese encephalitis virus

JEV was detected mainly in Taiwan (21.6%, n = 24), Korea (18%, n = 20) and Australia (15.3%, n = 17) (Fig. 1A). We found 23 mosquito species interacting with JEV: *Cx. vishnui* was the one most frequently found to be positive (IF = 1.20), followed by *Cx. tritaeniorhynchus* (IF = 1.17), *Cx. pipiens* and *Cx. annulus* (IF = 0.98) in Asia, while the most susceptible species in Oceania were *Cx. sitiens* and *Cx. gelidus* (IF = 0.71) (Supplementary Table 3).

The highest SMIR values were recorded in Asia in *Cx. rubithoracis* (62.38), *Cx. annulus* (47.68) and *Cx. tritaeniorhynchus* (28.16) (Fig. 2A and Supplementary Table 3), and *Cx. fuscocephala* had the highest estimated natural IR (Fig. 3A, Supplementary Table 3). Three genotypes were recorded: genotype I (strain VNKT/479/2007, VNKT/486/2007, and JEV Ishikawa12), genotype III (Tibet-Culex-JEV1-5), and genotype V (K12YJ1174). These were isolated in China, Vietnam, and Japan (Genotype I), Italy, China (Genotype III), and Korea (Genotype V) (Supplementary Table 2).

#### Usutu virus

Field studies on USUV have been conducted in Europe, Africa, and Asia, most of them in Italy (66.6%, n = 72), Czechoslovakia (11.1%, n = 12) and Slovakia (7.4%, n = 8) (Fig. 1A). Six species were reported to be susceptible to natural infection. *Cx. perexiguus* had the highest IF and SMIR (1.30) (Supplementary Table 3). In Africa, *Cx. antennatus* (IF = 1), and in Asia *Cx. pipiens* (IF = 1) were the most likely to be positive, while *Cx. pipiens* had the highest IR (5.19) (Fig. 3A, Supplementary Table 3). The recorded strains were USU181_09/USU090-10/USU173_09 (Italy) and USU/Croatia/Zagreb-102/2018 (Italy).

#### St. Louis encephalitis virus

The field studies on SLEV focused on North America (97.7%, n = 43) and Brazil (2.2%, n = 1). Three species were recorded interacting with this virus. *Cx. erraticus* had the highest IF (2.06), SMIR (2.06) and IR, followed by *Cx. quinquefasciatus* (North America) (IF = 0.73, SMIR = 1.97) (Fig. 2A, Supplementary Table 3).

The highest estimated IR of JES was for *Cx. pipiens* (Europe), which can be naturally infected with WNV and USUV, followed by *Cx. quinquefasciatus* (North America), which can be infected with WNV and SLEV (Fig. 3A).

### Experimental approach

Experimental studies were reported in 481 articles. After screening the titles, abstracts, and full texts, as well as opportunistic records, 95 articles remained for the analysis (Supplementary Fig. 2). From these we obtained 189 high quality observations of the TE of JES in 11 countries, 40 localities, and 21 species (Fig. 1B, Supplementary Table 1). The USA was the best represented country (54.4%, n = 103), followed by Germany (13.2%, n = 25) and Australia (12.6%, n = 24). There was, however, a notable lack of information on the vector competence of *Cx.* mosquitos for JES in many regions of the world, such as Central and South America, and Africa (Fig. 1B).

The most common means of infection was oral (94.8%, 395 observations), while the rest were intrathoracic. Intrathoracic infection bypasses the midgut barrier so is not considered natural infection. We therefore carried out the subsequent analyses using only the data on oral infection (Supplementary Table 4).

**Figure 4:**
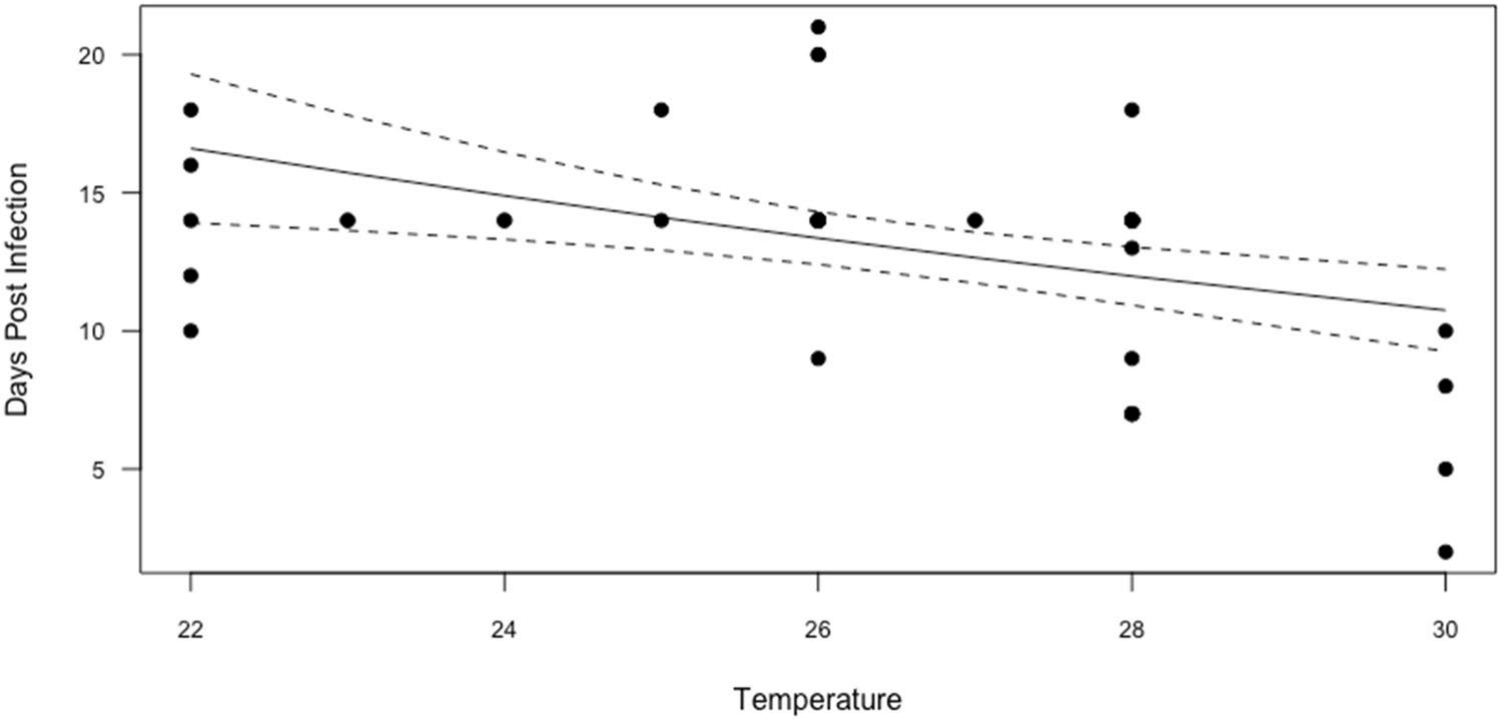
Relationship between temperature and Days Post Infection for WNV strain NY99.

We used a generalised linear model (GLM) for the statistical analysis, which was conducted only on the WNV dataset (strain NY99), the only one with sufficient observations for the purpose (n = 63). We did not find a significant effect of viral titre, temperature, or days post infection on TE. However, more data with a wide range of values is necessary to confirm these observations. On the other hand, we found that the Extrinsic Incubation Period (as DPI) was shorter at higher temperatures (Fig. 4 and Supplementary Table 5).

#### West Nile virus

Mosquito populations from many locations on all continents have been studied for their vector competence for this virus, particularly in the USA (60.3% of observations, n = 96), Germany (15.7%, n = 25) and Australia (6.9%) (Fig. 1B). Our bibliographic research revealed 21 species of *Cx.* with the ability to transmit WNV under laboratory conditions (Supplementary Table 6). *Cx. pipiens* (North America) and *Cx. tarsalis* were the most frequently studied species and were the most efficient in transmitting the virus (Transmission Frequency (TF) = 2.33) (Table1). *Cx. quinquefasciatus* had the highest TF (1.70) in Africa, *Cx. modestus* in Europe (TF = 1.32), and *Cx. annulirostris* and *Cx. quinquefasciatus* in Oceania (TF = 1.48) (Supplementary Table 6).

**Figure 5:**
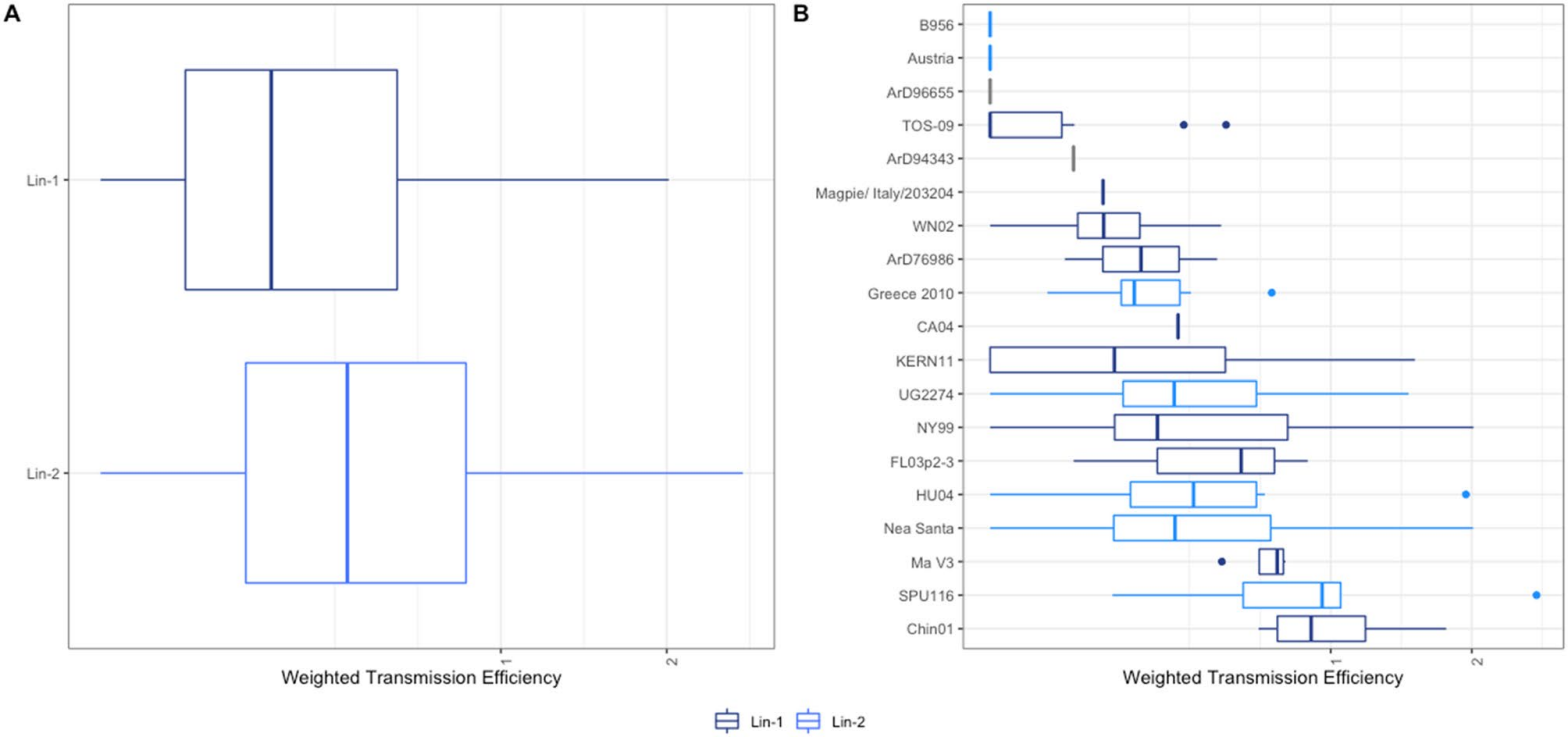
Box plots for WNV (**A**) lineages and (**B**) strains used to measure Weighted Transmission Rates.

Concerning Standardized Transmission Rates (STE) estimates, *Cx. quinquefasciatus* had the highest values in the USA (STE = 1.63), *Cx. pipiens* in Europe (0.90), *Cx. tritaeniorhynchus* in Asia (1.8), *Cx. neavei* in Africa (0.17) and *Cx. annulirostris* in Oceania (2.45) (Fig. 2B, Supplementary Table 6). We found 20 different strains of WNV tested. The TE of the various WNV strains vary considerably, but lineage 1 was more efficient than lineage 2. There were also more studies on the lineage 1 strains (n = 11), which exhibited high variation (Fig. 5).

#### Japanese encephalitis virus

JEV has been studied mainly in mosquito populations from France (45%, n = 20) and Australia (34%, n = 15), but also the United Kingdom, India, Taiwan, New Zealand, and the USA (Fig. 1B). Six mosquito species are capable of transmitting JEV. *Cx. pipiens* (Europe) had the highest TF (1.85), while *Cx. gelidus* had high values of STE (1.73) (Fig. 3B and Supplementary Table 6).

#### St. Louis encephalitis virus

Vector competence for SLEV has been studied in two countries: the USA (93.3%, n = 42) and Argentina (6.6%, n = 3), and 7 mosquito species have been investigated. *Cx. nigripalpus* was the most efficient in transmitting the virus (TF = 1.60), while *Cx. pipiens* had the highest STE (0.68) (Figs. 2B, 3B and Supplementary Table 6).

#### Usutu virus

Studies have also been conducted on the Usutu virus in mosquito populations in the USA (28.57%, n = 4), the United Kingdom (42.8%, n = 6) and Senegal (25%, n = 4), in particular on *Cx. neavei, Cx. pipiens* and *Cx. quinquefasciatus* (TF = 1). *Cx. neavei* also had the highest STE (0.79) (Fig. 3B).

We found reports of JES transmission under laboratory conditions in 22 *Cx.* species, and natural infections in 32 species (55.1% of the total sample) in the field. *Cx. pipiens* complex (biotypes *quinquefasciatus*, *pipiens, molestus and pallens*) was the most common vector accounting for 36.9% (n = 660) of the experimental observations and 25.7% (n = 1342) of the field observations. With both approaches, WNV was the most common flavivirus, accounting for 80.4% of the field observations and 86.7% of the experimental data (Fig. 1A,B). Only WNV, therefore, had enough observations to make comparison between the experimental and field data possible. We were able to compare 16 mosquito species and found a high positive correlation between TF and IF (R = 0.57, *p* = 0.02) (Fig. 7).

**Figure 6:**
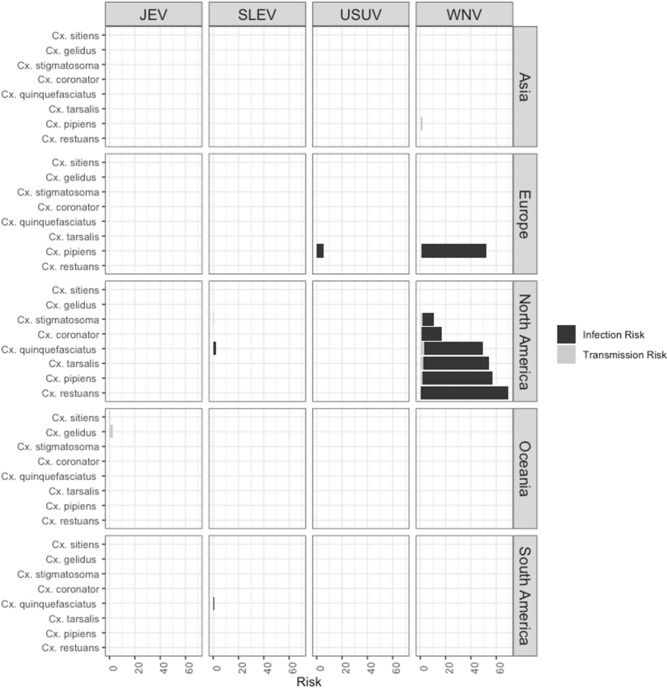
JES infection-transmission risk by continent and flavivirus.

In summary, we found that the species with the highest infection-transmission risk (IRT) for WNV was *Cx. restuans*, for USUV it was *Cx. pipiens* (Europe), for SLEV *Cx. quinquefasciatus* (North America), and for JEV *Cx. gelidus* (Oceania) (Fig. 6 and Supplementary Tables 2 and 6).

## Discussion

To the best of our knowledge, this study is the first review to explore on a worldwide scale the interactions among *Cx. mosquito* species and JES flaviviruses using both field and experimental data. We advise treating the results with some caution. First of all, the reported information is not definitive due to the scarcity of studies conducted in Africa, South America and Asia, and a lack of standardisation in laboratory and field methodologies. Furthermore, as we are dealing with multi-host, multi-vector disease systems, there is clearly variability among different geographical areas.

At least 30 mosquito species interact with JES viruses in natural conditions, and 23 species have been tested and confirmed as transmitters in the laboratory. Nonetheless, we are of the view that, although the information is incomplete, these results will be very important for researchers and policymakers designing surveillance and vector control strategies.

**Figure 7:**
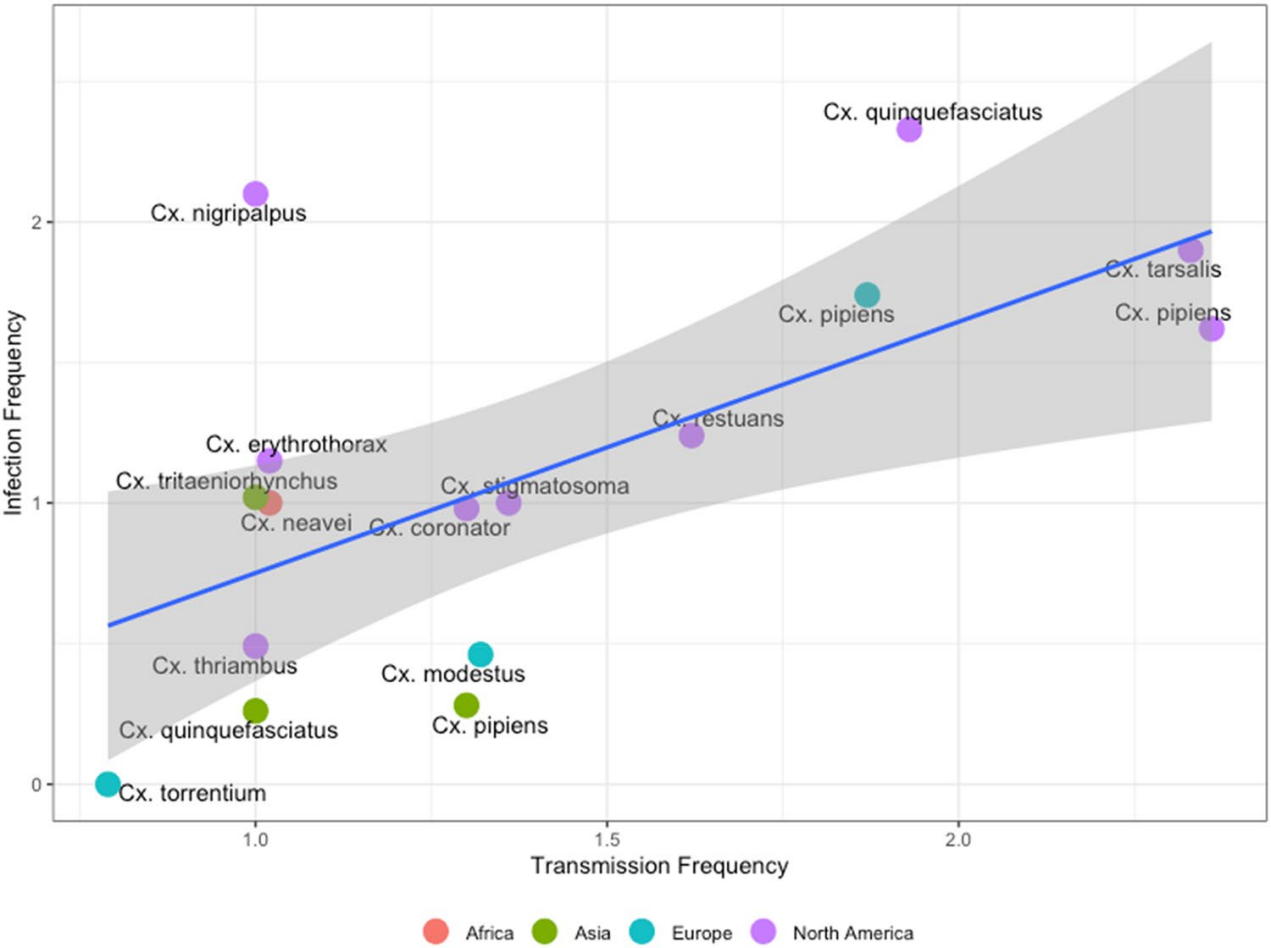
Comparison between Transmission Frequency and Infection Frequency for WNV.

*Cx. restuans* was the most efficient vector of WNV, with the highest ITR (Fig. 6). Our results agree with previous reports suggesting that *Cx. restuans* is a highly competent vector in the USA in both rural and urban areas^21^, and this, together with the fact that North American species were found to have the highest infection and transmission frequency, may explain the burden in the USA (Fig. 7)^8,22,23^. However, more data are needed to confirm this, as the many studies conducted in this country may have resulted in overestimation.

In Europe, the most important vector in the laboratory and in the field was *Cx. pipiens*, which is thought to be the principal vector of WNV^24^. *Cx. torrentium* also exhibited high transmission values in the laboratory, although its role needs to be confirmed with more field studies (Fig. 3A). Both species, which are ornithophilic, are abundant in Central Europe and have contributed to growing concerns in Europe over repeated outbreaks of WNV in recent years^25^. However, it should be borne in mind that *Cx. pipiens* and *Cx. torrentium* females can be only differentiated by PCR, so the reported data were probably mostly obtained from a mix of both species^26^.

In Africa, *Cx. neavei* had elevated values for both WNV and USUV, and its vector competence has been demonstrated in laboratory settings^27^. Experimental and field studies in Asia show that *Cx. tritaeniorhynchus* is to be considered a highly effective vector. In Oceania, *Cx. annulirostris, Cx. quinquefasciatus* and *Cx. gelidus* were found to be suitable vectors of WNV, but there is a lack of information on infection rates in the field. However, field data show that some species, such as *Cx. salinarius* and *Cx. bahamensis*, could be important potential vectors of WNV, and although some researchers suspect this to be the case, the hypothesis has yet to be tested in the laboratory^28^.

Our results highlight the uncertainty surrounding the vector competence of mosquito species and populations in Central and South America for WNV and SLEV^29^. For example, TE has been tested only for *Cx. nigripalpus* in Argentina and Honduras (Fig. 1). Field observations suggest that *Cx. interrogator* (Mexico), *Cx. mollis,* and *Cx. inflictus* (Guatemala) could be suitable vectors, but more studies are needed to fill these gaps^29,30^.

JEV has been tested only with Oceanic mosquito populations under experimental conditions. Although *Cx. annulirostris* was found to be a highly effective vector in the laboratory, this is not backed up by field data. *Cx. annulus, Cx. rubithoracis* and *Cx. tritaeniorhynchus* had the highest SMIR and IR values, but there are no supporting experimental studies. *Cx. tritaeniorhynchus* is an important vector in its endemic zones, but so far it has not been possible to breed and study this species in laboratory conditions^31^.

*Cx. quinquefasciatus* and *Cx. erraticus* were found to be potential competent vectors of SLEV (Supplementary Table 6), and it has been suggested that these species are the main vectors in the USA. Our database contains only one record in Central and South America, which concerns a single *Cx. quinquefasciatus* mosquito tested and found positive in Brazil^32^.

Finally, *Cx. pipiens* had the highest infection risk for USUV in experimental and field studies. This virus originated in Africa and has been studied under experimental conditions only with populations from Barkedji, Senegal, with positivity in *Cx. neavei* and *Cx. antennatus*^33^. In Europe, where Italy is the most frequently sampled country followed by Slovakia, *Cx. pipiens* is considered the most important vector. Some species, such as *Cx. modestus* and *Cx. territans*, have been found to be positive in the field, but the evidence is still weak for them.

Some limitations in our review are explained by the high variability in many of the factors that influence transmission and infection. Although the studies follow similar protocols, the results need to be interpreted individually as several conditions are specific, such as strain, viral load, microbiota, temperature, saliva collection, incubation time and phenotypic variation. In experimental studies, we found a few reports of viral titres in the salivary glands. The presence of a virus in mosquito saliva is not necessarily an indicator of the mosquito’s transmission potential, although viral titres are crucial to gaining a better understanding of transmission risk. The results are not the only determinant of the “real” infection-transmission risk in the field, because, as we have pointed out, vector competence is not the only determinant of transmission risk, as mosquito density, longevity and feeding preferences are also involved, as shown with regard to the estimations of vector capacity^11,13,14^.

Other limitations in our study regarding to the synthesis methodology is that for systematic reviews and macroecological studies include research and publication bias, which results in a biased view of the final estimates^34–36^. As an example, in our study, we observe that investigations focus on species a priori considerate competent vectors as *Cx. pipiens* (North America) sampled 167 times and underestimate others as 15 *Cx. mosquito* species were sampled only once.

In our view, positive results obtained by PCR from field samples do not mean these species are necessarily competent, but they can be a first indicator. It is crucial that: (1) each part of the mosquito’s body, i.e., thorax, legs and salivary glands, is tested individually, as they can spread the virus in different ways, such as infection, dissemination and transmission, respectively; (2) studies measure viral titres in salivary glands, as such data are considered more accurate and robust for drawing conclusions regarding vector competence, identifying new potentially vector competent mosquitoes, and consequently assessing infection risk^11,14^.

Our field estimates may be useful to focus on monitoring this particular *Cx.* mosquito species with high IF, MIR, and/or ITR values for each particular flavivirus and in a particular area. These *Cx. mosquito* species can be indicators on virus circulation and of the implication of a particular mosquito species on enzootic/epizootic/epidemic arboviral cycles, as a primary or secondary vector depending on MIR values. However, for vector capacity in the field other factors are also important. Among them, it is necessary to have a knowledge of its densities, feeding preferences, longevity, co-occurrences with human settlements or animal reservoirs and geographic ranges^37^.

Identification of mosquito species with high risk of natural infection in a particular area can help to target vector control strategies to their particular larval breeding sites (as particular containers) or the resting and blood-feeding adult habitats (as indoor or outdoor). Also, this knowledge could allow the application of specific control strategies for these species, such as the case of Sterile Insect Technique or Wolbachia-based strategies^10,37^. It is important to identify which mosquito species has high probabilities of natural infection and is likely to bite humans (or virus reservoirs) and also to be aware of the spatiotemporal overlap between mosquito and host densities^37^.

Regarding the laboratory approach, the identification of *Cx.* mosquito species with higher values in TE allows to evaluate the risk of transmission of an introduction of a virus in an area with a particular species or the introduction of an invasive vector in a new area^14^. Also, it can help to understand the contribution of each species in a particular geographical area to the burden of disease transmission. It can open avenues of research, as to understand the influence of other factors on vector competence (temperature, relative humidity, microbiome) and to test models of reducing disease transmission based on population replacement, as with Wolbachia-based vector control technique.

We conclude that on a worldwide scale, a combination of field and experimental data offers a better way of understanding natural infection and transmission risks between mosquito populations. Our analyses identified potentially competent vectors that could be important subjects for laboratory experiments and field studies. Finally, these results could be integrated into other analytical and modelling approaches aimed at estimating arboviral transmission risks in order to minimise transmission and reduce the health burden on humans and wildlife.

## Methods

### Bibliographic research

#### Literature search

Two separate, systematic literature reviews were conducted focusing on *Cx.* mosquito species and flaviviruses from the JES (WNV, JEV, USUV and SLEV): (1) field studies to identify viruses in mosquitoes using MIR data as the response variable; and (2) experimental studies of vector competence using TE values as the response variable. The search strategies are referred in the Supplementary Table 7. Both literature reviews were carried out in accordance with the guidelines of the Preferred Reporting Items for Systematic Reviews and Meta-Analyses Statement (PRISMA) using two online repositories: the Web of Science (WoS) platform (https://webofknowledge.com/) and PubMed (https://www.ncbi.nlm.nih.gov/pubmed/). We reviewed only English language, peer-reviewed papers published up to 4th October 2020. Additional papers were retrieved through bibliographic alerts set up by the review team, and opportunistic searches.

We carefully composed a set of appropriate search strings to run in WoS and PubMed and consensually retained the most efficient among them (Supplementary Figs. 1 and 2). Two independent reviewers screened them to ensure transparency and validity. However, it was often difficult to judge from the title and the abstract whether the content of an article was relevant, so many more articles were retained for further detailed analysis when agreement was not unanimous. Disagreements were resolved by consensus or consultation with a third reviewer.

#### PRISMA methodology

Potentially relevant materials obtained from all the repositories were combined in a single file and screened for duplicates. The documents retrieved were individually assessed following PRISMA guidelines: titles, then abstracts, and finally full text (Supplementary Figs. 1 and 2).

For the first review, i.e. field detection of JES flaviviruses in *Cx.* species, the inclusion criterion was field studies with reports of natural infections in mosquitos at the species level. Exclusion criteria were genus level observations and semi-field studies (i.e. in zoos). The PRISMA diagram is shown in Supplementary Fig. 1, while the database and article references are shown in Supplementary Table 2. As viral RNA detection depends on the screening method (broad, as with Pan-PCR, or specific, as with WNV-specific RT-PCR), we only considered results obtained separately for each flavivirus (e.g., WNV specific primers) or by sequencing positives, as in the case of the pan-flavivirus PCR protocols.

For the second review, i.e. experimental studies of the vector competence of *Cx.* species for JES flaviviruses, our inclusion criterion was estimated presence of virus in the saliva through Transmission Rates, Transmission Efficiency and viral titres. We also included as independent observations experiments investigating different temperatures, days post infection and viral loads. The exclusion criterion was studies that included several confounding factors when estimating vector competence, for example: (i) effects of the presence of *Wolbachia* infection, (ii) effects of insecticides, (iv) nutritional effects, (v) larva and their associated factors, (vi) virus mutations, and (vii) interactions with any kind of parasite or symbiont. However, this does not mean that these factors do not affect the mosquito’s transmission rate in natural conditions. The PRISMA diagram is shown in Supplementary Fig. 2, the database and references of the articles are shown in Supplementary Table 4.

The database for the review of the field studies was constructed by extracting the following variables: (i) bibliographic reference, (ii) country, state/province, and locality of the study, (iii) mosquito species, (iv) screening method, (v) number of mosquitoes tested/number of positive mosquitoes, and (vi) number of pools tested/number of positive pools, with minimum infection rates and/or maximum likelihood estimation as our response variable. These measures are common surveillance indicators used to assess the risk of transmitting viruses to other vertebrates, including humans^38^. In both cases (experimental and field reviews), observations from the same article and on the same mosquito species but tested in different localities or sampling years were considered independent data. Observations reported as *Cx.* spp. were excluded.

For the review of the experimental studies, we created a database (Supplementary Table 4) with the following information: (i) bibliographic reference, (ii) country, state/province and locality, (iii) mosquito species, (iv) number of individuals tested, (v) infection methodology, (vi) virus lineage and strain, (vii) temperature, (viii) days post incubation, and (ix) diagnostic method. Observations from studies reporting multiple TEs from separate experiments using different temperatures, strains, and days post infection were considered independent data in our analyses.

We evaluated the reliability of the methodology and response values reported by the authors. Determining the relevance of each reported value is not straightforward and can be affected by a high level of subjectivity. Instead, we assessed objectively whether the estimation method was documented and traceable. The quality of the estimations was classified as: (i) high, when the numbers of individuals tested for TE or MIR were specified, (ii) medium, when only TE or MIR were specified, and iii) low, when TE and MIR were merely reported as positive or negative^39^. The main analysis was carried out only with the high-quality data, because large sample sizes are essential for statistical precision^40^.

### Data analysis

We used TE as the main indicator of vector competence**,** which can, however, be influenced by four main factors: temperature (*T*), viral titre of infection (*VT*), days post infection (*DPI*), and virus strain (*S*). We therefore performed generalised linear models with negative binomial distribution that included these factors as variables and the number of mosquitoes tested by observation as an offset variable. We then tested the collinearity between all the independent variables. Akaike’s information criterion (AIC) was used to select the best model, and the explained deviance was calculated as (null model deviance-residual deviance)/null model deviance. The WNV flavivirus was the one with sufficient observations to perform these models.

$$TE\sim T + VT + DPI + S$$

Due to the high variation among studies, to determine sampling effort we weighted the TE and MIR estimates by multiplying the log^10^ of the number of individuals tested per species by each observation. We therefore added one unit to the observations comprising only 1 sampled individual^18^.

$$WTE = TE \times (1 + \log^{10} \,\,number\,\, of\,\, individuals \,\,tested)$$

$$WMIR = MIR \times \left( {1 + \log^{10 } \,\,number \,\,of\,\, individuals\,\, tested} \right)$$

Having obtained the weighted score for each observation, we calculated the standardised transmission efficiency (STE) and standardised minimum infection rates (SMIR) (Table 1). We then calculated the mean TE and MIR values by mosquito species and carried out a second weighting by the number of times each species was studied^18^.

$$STE = mean\,\, TE \times (1 + log^{10} \,\,number \,\,of\,\, experiments)$$

$$SMIR = mean\,\, MIR \times \left( {1 + log^{10 } \,\,number \,\,of\,\, experiments} \right)$$

We also calculated the transmission frequency (TF) by mosquito species (Supplementary Table 6) using the low-quality data from the experimental studies (Table 1).

$$TF = \frac{{times\,\, positive\,\, \left( {saliva} \right)}}{number \,\,of\,\, infections} \times \left( {1 + log^{10 } \,\, number \,\,of\,\, experiments} \right)$$

Similarly for the field data, we estimated the infection frequency (IF) for each mosquito species (Supplementary Table 3) as follows:

$$IF = \frac{{times \,\,positive \,\,\left( {infected} \right)}}{number\,\, of\,\,observations} \times \left( {1 + log^{10 } \,\,number\,\, of \,\,observations} \right)$$

We also calculated the virus infection-transmission risk (ITR) by modifying the risk equation, i.e. as the product of TF and STE, while virus infection risk was estimated as the product of IF and SMIR for each mosquito species and flavivirus studied^41^ (Table 1).

Finally, we carried out a Spearman coefficient correlation to quantify the strength of the association between TF and IF. We performed all the analyses and produced the figures with the R software version 4.1.2 using the follow packages (tidyverse, dplyr, ape, gapminder, ggplot2, ggpubr, tidyr, ggrepel, ggthemes, hrbrthemes, MASS, maps, and mapdata).

## Supplementary Information

Supplementary Information 1.

Supplementary Information 2.

Supplementary Information 3.

## Data Availability

All data generated or analysed during this study are included in this published article (and its supplementary information files).
