# Supplementary material for: Worldwide transmission and infection risk of mosquito vectors of West Nile, St. Louis encephalitis, Usutu and Japanese encephalitis viruses: a systematic review
Source: Sci Rep. 2023 Jan 6;13:308. doi: 10.1038/s41598-022-27236-1 (PMC9822987; doi:10.1038/s41598-022-27236-1)
Supplement: Supplementary file 1 — Supplementary Information 1. [file 41598_2022_27236_MOESM1_ESM.docx]

**Worldwide Transmission and Infection Risk of mosquito vectors of West Nile, St. Louis Encephalitis, Usutu and Japanese Encephalitis viruses: A systematic review.**

Tolsá-García María José*, Magdalena L. Wehmeyer, Renke Lühken, David Roiz

**SUPPLEMENTARY MATERIAL**

Records after duplicates removed
(n = 301)

Records identified through database searching
PubMed n =198

Records identified through database searching
WoS n = 260

Additional records identified through other sources
(n = 57)

## Eligibility

## Screening

## Included

## Identification

Studies included in the qualitative analyse

(n = 130)

Studies included after full-text screening
(n = 73)

Records after screening by abstract (n =140)

Records after screening by title
(n = 178)

**Supplementary Figure 1.** Flow diagram of the process of article search of field studies based on the PRISMA protocol (Liberati et al., 2009).

| **FLAVIVIRUS** | **DESCRIPTION** | **FIELD APPROACH** | **EXPERIMENTAL APPROACH** |
| --- | --- | --- | --- |
| WNV | No. observations | 1079 | 620 |
|  | High quallity observations | 540 | 203 |
|  | Number mosquito species tested | 41 | 21 |
|  | Number of mosquito species interacting with viruses | 24 | 20 |
| JEV | No. observations | 111 | 44 |
|  | High quallity observations | 80 | 21 |
|  | Number mosquito species tested | 23 | 6 |
|  | Number of mosquito species interacting with viruses | 11 | 6 |
| USUV | No. observations | 108 | 14 |
|  | High quallity observations | 75 | 9 |
|  | Number mosquito species tested | 14 | 7 |
|  | Number of mosquito species interacting with viruses | 4 | 7 |
| SLEV | No. observations | 44 | 45 |
|  | High quallity observations | 38 | 7 |
|  | Number mosquito species tested | 6 | 3 |
|  | Number of mosquito species interacting with viruses | 3 | 3 |

**Supplementary Table 1.** Number of observations and mosquito species for both approaches

**Supplementary Table 2.** Field database and their references for JES **(Excel)**

| **FLAVIVIRUS** | **MOSQUITO SPECIES** | **INFECTION FREQUENCY** | | | | **MIR** | | | | **VIRUS INFECTION RISK** |  |
| --- | --- | --- | --- | --- | --- | --- | --- | --- | --- | --- | --- |
|  |  | **CONTINENT** | **TIMES TESTED** | **TIMES POSITIVE** | **POSITIVITY** | **CONTINENT** | **TIMES TESTED** | **MIR MEAN** | **STANDARIZED MINIMUN INFECTION RATES** |  |  |
|  |  |  |  |  |  |  |  |  |  |  |  |
| WNV | *Cx. antennatus* | Africa | 1 | 1 | 1.00 | NA | NA | NA | NA | NA |  |
|  | *Cx. atratus* | North America | 5 | 0 | 0.00 | North America | 4 | 0 | 0.00 | 0.00 |  |
|  | *Cx. bahamensis* | North America | 6 | 4 | 1.19 | North America | 4 | 13.52 | 21.66 | 25.68 |  |
|  | *Cx. chidesteri* | North America | 3 | 0 | 0.00 | North America | 3 | 0 | 0.00 | 0.00 |  |
|  | *Cx. coronator* | North America | 3 | 2 | 0.98 | North America | 2 | 12.53 | 16.30 | 16.05 |  |
|  | *Cx. declarator* | North America | 1 | 0 | 0.00 | North America | 1 | 0 | 0.00 | 0.00 |  |
|  | *Cx. erraticus* | North America | 29 | 10 | 0.85 | North America | 24 | 4.78 | 11.38 | 9.66 |  |
|  | *Cx. erythrothorax* | North America | 20 | 10 | 1.15 | North America | 11 | 0.1 | 0.20 | 0.23 |  |
|  | *Cx. habilitator* | North America | 3 | 1 | 0.49 | North America | 3 | 12.75 | 18.83 | 9.27 |  |
|  | *Cx. hortensis* | Asia | 2 | 0 | 0.00 | Asia | 1 | 0 | 0.00 | 0.00 |  |
|  | *Cx. hortensis* | Europe | 8 | 0 | 0.00 | Europe | 3 | 0 | 0.00 | 0.00 |  |
|  | *Cx. impudicus* | Europe | 1 | 0 | 0.00 | Europe | 1 | 0 | 0.00 | 0.00 |  |
|  | *Cx. iolambdis* | North America | 4 | 0 | 0.00 | North America | 4 | 0 | 0.00 | 0.00 |  |
|  | *Cx. janitor* | North America | 3 | 0 | 0.00 | North America | 3 | 0 | 0.00 | 0.00 |  |
|  | *Cx. martinii* | Europe | 1 | 0 | 0.00 | Europe | 1 | 0 | 0.00 | 0.00 |  |
|  | *Cx. mimeticus* | Asia | 2 | 0 | 0.00 | NA | NA | NA | NA | NA |  |
|  | *Cx. mimeticus* | Europe | 1 | 0 | 0.00 | Europe | 1 | 0 | 0.00 | 0.00 |  |
|  | *Cx. modestus* | Europe | 33 | 6 | 0.46 | Europe | 24 | 0 | 0.00 | 0.00 |  |
|  | *Cx. mulrennani* | America | 1 | 0 | 0.00 | North America | 1 | 0 | 0.00 | 0.00 |  |
|  | *Cx. neavei* | Africa | 1 | 1 | 1.00 | NA | NA | NA | NA | NA |  |
|  | *Cx. nigripalpus* | North America | 38 | 31 | 2.10 | North America | 23 | 6.8 | 16.06 | 33.80 |  |
|  | *Cx. peccator* | North America | 3 | 0 | 0.00 | North America | 2 | 0 | 0.00 | 0.00 |  |
|  | *Cx. perexiguus* | Europe | 2 | 1 | 0.65 | Europe | 1 | 0 | 0.00 | 0.00 |  |
|  | *Cx. perexiguus* | Asia | 2 | 0 | 0.00 | NA | NA | NA | NA | NA |  |
|  | *Cx. perexiguus* | Africa | 1 | 1 | 1.00 | NA | NA | NA | NA | NA |  |
|  | *Cx. perfuscus* | Africa | 1 | 1 | 1.00 | NA | NA | NA | NA | NA |  |
|  | *Cx. pilosus* | North America | 1 | 0 | 0.00 | North America | 1 | 0 |  |  |  |
|  | *Cx. pipiens* | Africa | 9 | 4 | 0.87 | Africa | 1 | 20.45 | 20.45 | 17.76 |  |
|  | *Cx. pipiens* | North America | 167 | 84 | 1.62 | North America | 105 | 11.23 | 33.93 | 55.00 |  |
|  | *Cx. pipiens* | Asia | 16 | 2 | 0.28 | Asia | 11 | 0 | 0.00 | 0.00 |  |
|  | *Cx. pipiens* | Europe | 88 | 52 | 1.74 | Europe | 29 | 11.88 | 29.25 | 50.90 |  |
|  | *Cx. poicilipes* | Africa | 1 | 1 | 1.00 | NA | NA | NA | NA | NA |  |
|  | *Cx. pseudovishnui* | Asia | 4 | 1 | 0.40 | NA | NA | NA | NA | NA |  |
|  | *Cx. quinquefasciatus* | North America | 122 | 92 | 2.33 | North America | 61 | 7.12 | 19.83 | 46.16 |  |
|  | *Cx. quinquefasciatus* | Africa | 1 | 1 | 1.00 | NA | NA | NA | NA | NA |  |
|  | *Cx. quinquefasciatus* | Asia | 7 | 1 | 0.26 | NA | NA | NA | NA | NA |  |
|  | *Cx. restuans* | North America | 74 | 32 | 1.24 | North America | 44 | 21.19 | 56.01 | 69.50 |  |
|  | *Cx. sitiens* | Asia | 1 | 0 | 0.00 | NA | NA | NA | NA | NA |  |
|  | *Cx. salinarius* | North America | 51 | 33 | 1.75 | North America | 22 | 5.54 | 12.98 | 22.74 |  |
|  | *Cx. stigmatosoma* | North America | 21 | 9 | 1.00 | North America | 17 | 4.15 | 9.26 | 9.21 |  |
|  | *Cx. taeniopus* | North America | 3 | 0 | 0.00 | North America | 3 | 0 | 0.00 | 0.00 |  |
|  | *Cx. tarsalis* | North America | 120 | 74 | 1.90 | North America | 94 | 9.24 | 27.47 | 52.16 |  |
|  | *Cx. territans* | Europe | 5 | 0 | 0.00 | Europe | 4 | 0 | 0.00 | 0.00 |  |
|  | *Cx. territans* | North America | 16 | 0 | 0.00 | North America | 14 | 0 | 0.00 | 0.00 |  |
|  | *Cx. theileri* | Asia | 12 | 0 | 0.00 | Asia | 10 | 0 | 0.00 | 0.00 |  |
|  | *Cx. theileri* | Europe | 10 | 0 | 0.00 | Europe | 2 | 0 | 0.00 | 0.00 |  |
|  | *Cx. thriambus* | North America | 3 | 1 | 0.49 | North America | 2 | 0 | 0.00 | 0.00 |  |
|  | *Cx. torrentium* | Europe | 1 | 0 | 0.00 | Europe | 1 | 0 | 0.00 | 0.00 |  |
|  | *Cx. tritaeniorhynchus* | Europe | 1 | 0 | 0.00 | Europe | 1 | 0 | 0.00 | 0.00 |  |
|  | *Cx. tritaeniorhynchus* | Africa | 1 | 1 | 1.00 | NA | NA | NA | NA | NA |  |
|  | *Cx. tritaeniorhynchus* | Asia | 5 | 3 | 1.02 | NA | NA | NA | NA | NA |  |
|  | *Cx. univittatus* | Europe | 6 | 2 | 0.59 | Europe | 1 | 0.11 | 0.11 | 0.07 |  |
|  | *Cx. vishnui* | Asia | 4 | 2 | 0.80 | NA | NA | NA | NA | NA |  |
|  | *Cx. whitmorei* | Asia | 4 | 1 | 0.40 | NA | NA | NA | NA | NA |  |
| JEV | Cx. annulus | Asia | 3 | 2 | 0.98 | Asia | 3 | 32.28 | 47.68 | 46.95 |  |
|  | Cx. bitaeniorhynchus | Asia | 6 | 1 | 0.30 | Asia | 4 | 2.47 | 3.96 | 1.17 |  |
|  | Cx. brevipalpis | Asia | 1 | 0 | 0.00 | Asia | 1 | 0 | 0.00 | 0.00 |  |
|  | Cx. fuscanus | Asia | 2 | 0 | 0.00 | Asia | 2 | 0 | 0.00 | 0.00 |  |
|  | Cx. fuscocephala | Asia | 4 | 2 | 0.80 | Asia | 3 | 11.56 | 17.08 | 13.68 |  |
|  | Cx. gelidus | Asia | 2 | 0 | 0.00 | Asia | 1 | 0 | 0.00 | 0.00 |  |
|  | Cx. gelidus | Oceania | 8 | 3 | 0.71 | Oceania | 8 | 0 | 0.00 | 0.00 |  |
|  | Cx. hayashii | Asia | 1 | 0 | 0.00 | NA | NA | NA | NA | NA |  |
|  | Cx. inatomii | Asia | 3 | 0 | 0.00 | Asia | 2 | 0 | 0.00 | 0.00 |  |
|  | Cx. infula | Asia | 1 | 0 | 0.00 | Asia | 1 | 0 | 0.00 | 0.00 |  |
|  | Cx. mimeticus | Asia | 2 | 0 | 0.00 | Asia | 1 | 0 | 0.00 | 0.00 |  |
|  | Cx. murrelli | Asia | 1 | 0 | 0.00 | Asia | 1 | 0 | 0.00 | 0.00 |  |
|  | Cx. nigropunctatus | Asia | 1 | 0 | 0.00 | Asia | 1 | 0 | 0.00 | 0.00 |  |
|  | Cx. orientalis | Asia | 2 | 0 | 0.00 | Asia | 2 | 0 | 0.00 | 0.00 |  |
|  | Cx. pipiens | Asia | 3 | 2 | 0.98 | Asia | 3 | 7.55 | 11.15 | 10.98 |  |
|  | Cx. pseudovishnui | Asia | 1 | 0 | 0.00 | Asia | 1 | 0 | 0.00 | 0.00 |  |
|  | Cx. quinquefasciatus | Asia | 5 | 1 | 0.34 | Asia | 5 | 1.34 | 2.28 | 0.77 |  |
|  | Cx. rubensis | Asia | 1 | 0 | 0.00 | NA | NA | NA | NA | NA |  |
|  | Cx. rubithoracis | Asia | 2 | 1 | 0.65 | Asia | 2 | 47.95 | 62.38 | 40.58 |  |
|  | Cx. sitiens | Asia | 3 | 1 | 0.49 | Asia | 3 | 2.14 | 3.16 | 1.56 |  |
|  | Cx. sitiens | Oceania | 8 | 3 | 0.71 | Oceania | 8 | 0 | 0.00 | 0.00 |  |
|  | Cx. tritaeniorhynchus | Asia | 27 | 13 | 1.17 | Asia | 24 | 11.83 | 28.16 | 32.96 |  |
|  | Cx. vagans | Asia | 2 | 0 | 0.00 | Asia | 1 | 0 | 0.00 | 0.00 |  |
|  | Cx. vishnui | Asia | 4 | 3 | 1.20 | Asia | 2 | 2.72 | 3.54 | 4.25 |  |
|  | Cx. whitmorei | Asia | 2 | 0 | 0.00 | Asia | 1 | 0 | 0.00 | 0.00 |  |
| USUV | *Cx. antennatus* | Africa | 1 | 1 | 1.00 | NA | NA | NA | NA | NA |  |
|  | Cx. hortensis | Europe | 3 | 0 | 0.00 | Europe | 3 | 0 | 0.00 | 0.00 |  |
|  | Cx. impudicus | Europe | 1 | 0 | 0.00 | Europe | 1 | 0 | 0.00 | 0.00 |  |
|  | Cx. mimeticus | Europe | 1 | 0 | 0.00 | Europe | 1 | 0 | 0.00 | 0.00 |  |
|  | Cx. modestus | Europe | 24 | 0 | 0.00 | Europe | 23 | 0 | 0.00 | 0.00 |  |
|  | Cx. neavei | Africa | 1 | 1 | 1.00 | NA | NA | NA | NA | NA |  |
|  | Cx. perexiguus | Europe | 2 | 2 | 1.30 | NA | NA | NA | NA | NA |  |
|  | Cx. perfuscus | Africa | 1 | 0 | 0.00 | NA | NA | NA | NA | NA |  |
|  | Cx. pipiens | Europe | 44 | 16 | 0.96 | Europe | 43 | 2.05 | 5.40 | 5.19 |  |
|  | Cx. pipiens | Asia | 1 | 1 | 1.00 | NA | NA | NA | NA | NA |  |
|  | Cx. poicilipes | Africa | 1 | 0 | 0.00 | NA | NA | NA | NA | NA |  |
|  | Cx. quinquefasciatus | Africa | 1 | 0 | 0.00 | NA | NA | NA | NA | NA |  |
|  | Cx. territans | Europe | 3 | 0 | 0.00 | Europe | 3 | 0 | 0.00 | 0.00 |  |
|  | Cx. theileri | Europe | 2 | 0 | 0.00 | Europe | 1 | 0 | 0.00 | 0.00 |  |
|  | Cx. tritaeniorhynchus | Africa | 1 | 0 | 0.00 | NA | NA | NA | NA | NA |  |
| SLEV | Cx. erraticus | North America | 1 | 1 | 1.00 | North America | 1 | 2.06 | 2.06 | 2.06 |  |
|  | Cx. erythrothorax | North America | 6 | 0 | 0.00 | North America | 6 | 0 | 0.00 | 0.00 |  |
|  | Cx. pipiens | North America | 2 | 0 | 0.00 | NA | NA | NA | NA | NA |  |
|  | Cx. quinquefasciatus | North America | 19 | 10 | 1.20 | North America | 18 | 0.73 | 1.65 | 1.97 |  |
|  | Cx. quinquefasciatus | South America | 1 | 1 | 1.00 | South America | 1 | 0.29 | 0.29 | 0.29 |  |
|  | Cx. stigmatosoma | North America | 6 | 0 | 0.00 | North America | 6 | 0 | 0.00 | 0.00 |  |
|  | Cx. tarsalis | North America | 9 | 2 | 0.43 | North America | 6 | 0 | 0.00 | 0.00 |  |

**Supplementary Table 3.** Infection frequency to natural infections and MIR values for JES.

Records after duplicates removed
(n = 481)

Records identified through database searching
PubMed n =159

Records identified through database searching
WoS n = 462

Additional records identified through other sources
(n = 27)

## Eligibility

## Screening

## Included

## Identification

## ation

Studies included in the qualitative analyse

(n = 95)

Studies included after full-text screening
(n =68)

Records after screening by abstract (n =72)

Records after screening by title
(n = 107)

**Supplementary Figure *2.*** Flow diagram of the process of article search of experimental studies based on the PRISMA protocol (Liberati et al., 2009).

**Supplementary Table 4.** Experimental database and their references for JES **(Excel)**

|  | **VARIABLE** | **ESTIMATE** | **S.E.** | **z** | **p** |
| --- | --- | --- | --- | --- | --- |
| **Transmission Efficiency** | Viral titer | 6.489e-11 | 2.501e-11 | 2.595 | 0.00946 * |
|  | | | | | |
| **Transmission Efficiency** | Temperature | 3.778e-02 | 4.931e-02 | 0.766 | 0.44364 |
|  | Viral titer | 6.548e-11 | 2.495e-11 | 7.674 | 0.00868* |
|  | | | | | |
| **Transmission Efficiency** | Temperature | 5.987e-02 | 5.130e-02 | 1.167 | 0.2432 |
|  | Viral titer | 6.370e-11 | 2.483e-11 | 2.565 | 0.0103 * |
|  | Days Post Infection | 3.451e-02 | 2.112e-02 | 1.634 | 0.1023 |

**Supplementary Table 5.** Experimental database and their references for JES

**Supplementary Table 6.** Transmission frequency and transmission rates in experimental studies

| **FLAVIVIRUS** | **MOSQUITO SPECIES** | **TRANSMISSION FREQUENCY** | | | | **TRANSMISSION EFFICIENCY** | | | | **VIRUS TRANSMISSION RISK** |
| --- | --- | --- | --- | --- | --- | --- | --- | --- | --- | --- |
|  |  | **CONTINENT** | **TIMES TESTED** | **TIMES POSITIVE** | **POSITIVITY** | **CONTINENT** | **TIMES TESTED** | **TRANSMISSION EFFICIENCY (MEAN)** | **STANDARIZED TRANMISSION EFFICIENCY** |  |
| **WNV** | *Cx. annulirostris* | Oceania | 3 | 3 | 1.48 | Oceania | 3 | 1.66 | 2.45 | 3.62 |
|  | *Cx. australicus* | Oceania | 1 | 0 | 0.00 | Oceania | 1 | 0 | 0.00 | 0.00 |
|  | *Cx. bitaeniorhynchus* | Oceania | 1 | 1 | 1.00 | Oceania | 1 | 0.98 | 0.98 | 0.98 |
|  | *Cx. coronator* | North America | 5 | 5 | 1.70 | North America | 5 | 0.31 | 0.53 | 0.89 |
|  | *Cx. erythrothorax* | North America | 5 | 3 | 1.02 | NA | NA | NA | NA | NA |
|  | *Cx. gelidus* | Asia | 3 | 3 | 1.48 | NA | NA | NA | NA | NA |
|  | *Cx. gelidus* | Oceania | 1 | 1 | 1.00 | Oceania | 1 | 1.77 | 1.77 | 1.77 |
|  | *Cx. modestus* | Asia | 1 | 1 | 1.00 | Asia | 1 | 0.65 | 0.65 | 0.65 |
|  | *Cx. modestus* | Europe | 7 | 5 | 1.32 | Europe | NA | NA | NA | NA |
|  | *Cx. molestus* | North America | 2 | 2 | 1.30 | NA | NA | NA | NA | NA |
|  | *Cx. neavei* | Africa | 4 | 2 | 0.80 | Africa | 4 | 0.17 | 0.27 | 0.22 |
|  | *Cx. nigripalpus* | North America | 1 | 1 | 1.00 | North America | NA | NA | NA | NA |
|  | *Cx. nigripalpus* | South America | 5 | 3 | 1.02 | South America | NA | NA | NA | NA |
|  | *Cx. pervigilans* | Oceania | 1 | 1 | 1.00 | Oceania | 1 | 0.08 | 0.08 | 0.08 |
|  | *Cx. pipiens* | North America | 111 | 85 | 2.33 | North America | 40 | 0.3 | 0.78 | 1.82 |
|  | *Cx. pipiens* | South America | 2 | 1 | 0.65 | South America | 1 | 0.09 | 0.09 | 0.06 |
|  | *Cx. pipiens* | Asia | 2 | 2 | 1.30 | Asia | 1 | 1.07 | 1.07 | 1.39 |
|  | *Cx. pipiens* | Europe | 69 | 26 | 1.07 | Europe | 32 | 0.36 | 0.90 | 0.96 |
|  | *Cx. quinquefasciatus* | Africa | 5 | 5 | 1.70 | Africa | 1 | 0.07 | 0.07 | 0.12 |
|  | *Cx. quinquefasciatus* | North America | 56 | 41 | 2.01 | North America | 27 | 0.67 | 1.63 | 3.28 |
|  | *Cx. quinquefasciatus* | Asia | 1 | 1 | 1.00 | Asia | 2 | 0.77 | 1.00 | 1.00 |
|  | *Cx. quinquefasciatus* | Oceania | 3 | 3 | 1.48 | Oceania | 3 | 1.2 | 1.77 | 2.62 |
|  | *Cx. quinquefasciatus* | South America | 2 | 2 | 1.30 | South America | 2 | 0.16 | 0.21 | 0.27 |
|  | *Cx. restuans* | North America | 28 | 19 | 1.66 | North America | 1 | 0.18 | 0.18 | 0.30 |
|  | *Cx. sitiens* | Oceania | 3 | 2 | 0.98 | Oceania | 3 | 0.13 | 0.19 | 0.19 |
|  | *Cx. stigmatosoma* | North America | 5 | 4 | 1.36 | North America | 2 | 0.95 | 1.24 | 1.68 |
|  | *Cx. tarsalis* | North America | 68 | 56 | 2.33 | North America | 20 | 0.39 | 0.90 | 2.09 |
|  | *Cx. thriambus* | North America | 1 | 1 | 1.00 | North America | 1 | 0.83 | 0.83 | 0.83 |
|  | *Cx. torrentium* | Europe | 7 | 3 | 0.79 | Europe | 7 | 0.15 | 0.28 | 0.22 |
|  | *Cx. tritaeniorhynchus* | Asia | 1 | 1 | 1.00 | Asia | 1 | 1.8 | 1.80 | 1.80 |
|  | *Cx. univittatus* | Africa | 5 | 4 | 1.36 | Africa | NA | NA | NA | NA |
|  | *Cx. vansomerni* | Africa | 4 | 4 | 1.60 | Africa | NA | NA | NA | NA |
| **JEV** | *Cx. annulirostris* | Oceania | 6 | 5 | 1.48 | Oceania | 6 | 0.56 | 1.00 | 1.48 |
|  | *Cx. gelidus* | Oceania | 2 | 2 | 1.30 | Oceania | 2 | 1.33 | 1.73 | 2.25 |
|  | *Cx. pipiens* | Asia | 1 | 1 | 1.00 | NA | NA | NA | NA | NA |
|  | *Cx. pipiens* | Europe | 7 | 7 | 1.85 | Europe | NA | NA | NA | NA |
|  | *Cx. quinquefasciatus* | Oceania | 2 | 1 | 0.65 | Oceania | 2 | 0.1 | 0.13 | 0.08 |
|  | *Cx. quinquefasciatus* | North America | 1 | 0 | 0.00 | North America | 1 | 0 | 0.00 | 0.00 |
|  | *Cx. sitiens* | Oceania | 3 | 3 | 1.48 | Oceania | 3 | 0.36 | 0.53 | 0.79 |
|  | *Cx. tritaeniorhynchus* | Asia | 1 | 1 | 1.00 | NA | NA | NA | NA | NA |
| **SLEV** | *Cx. inferior* | South America | 1 | 1 | 1.00 | South America | 1 | 0.47 | 0.47 | 0.47 |
|  | *Cx. nigripalpus* | North America | 4 | 4 | 1.60 | NA | NA | NA | NA | NA |
|  | *Cx. pipiens* | North America | 1 | 1 | 1.00 | North America | 1 | 0.68 | 0.68 | 0.68 |
|  | *Cx. quinquefasciatus* | North America | 3 | 3 | 1.48 | North America | 1 | 0.18 | 0.18 | 0.27 |
|  | *Cx. quinquefasciatus* | South America | 1 | 1 | 1.00 | South America | 1 | 0.46 | 0.46 | 0.46 |
|  | *Cx. saltanensis* | South America | 1 | 1 | 1.00 | South America | 1 | 0.35 | 0.35 | 0.35 |
|  | *Cx. stigmatosoma* | North America | 1 | 1 | 1.00 | North America | 1 | 0.44 | 0.44 | 0.44 |
|  | *Cx. tarsalis* | North America | 9 | 7 | 1.52 | North America | 1 | 0.07 | 0.07 | 0.11 |
| **USUV** | *Cx. neavei* | Africa | 1 | 1 | 1.00 | Africa | 1 | 0.79 | 0.79 | 0.79 |
|  | *Cx. pipiens* | North America | 1 | 1 | 1.00 | North America | 1 | 0.34 | 0.34 | 0.34 |
|  | *Cx. pipiens* | Europe | 6 | 1 | 0.30 | Europe | 6 | 0.01 | 0.02 | 0.01 |
|  | *Cx. quinquefasciatus* | North America | 1 | 1 | 1.00 | North America | 1 | 0.32 | 0.32 | 0.32 |

| **ELECTRONIC DATABASES** | **ALTERNATIVES** |
| --- | --- |
| **FIELD STUDIES** | |
| Web of Science | [title/abstract] (flavivirus* OR West Nile OR Usutu OR St. Louis encephalitis OR Japanese encephalitis) AND (mosquito*) AND (Culex) NOT (ticks) NOT (review*) AND (PCR*) |
| Pubmed | [title/abstract] (“flavivirus” OR “West Nile” OR “Usutu” OR “St. Louis encephalitis” OR “Japanese encephalitis”) AND (“mosquito”) AND (“Culex”) NOT (“ticks”) NOT (“review”) AND “PCR” |
| **EXPERIMENTAL STUDIES** | |
| Web of Science | [title/abstract] ("vector competence" OR "transmission efficiency" OR "transmission potential" OR "replication potential") AND (flavivirus* OR West Nile OR Usutu OR St. Louis encephalitis OR Japanese encephalitis) AND Culex AND mosquito* |
| Pubmed | [title/abstract] ("vector competence" OR "transmission efficiency" OR "transmission potential" OR "replication potential") AND (flavivirus* OR west nile OR usutu OR St. Louis encephalitis OR Japanese encephalitis) AND Culex AND mosquito* |

**Supplementary Table 7.** Search terms
